# The Treatment of Acute Chorea

**Published:** 1892-12-17

**Authors:** 


### SUSSEX COUNTY HOSPITAL.

### The Treatment of Acute Chorea.

There are few cases in the medical wards of the Sussex County Hospital which give rise to more anxiety than those of the disease under consideration.

The victims are more often girls about the age of puberty. The pathology of this strange "insanity of the muscles" is as yet involved in obscurity, but the malady is generally considered to belong to the dark category of the epilepsies and insanities.

The treatment adopted is, therefore, based on a recognition of the prodigal discharge of nervous energy accompanying the disease, and of the consequent corresponding exhaustion of the nerve elements. Every effort is made:—

1. To curb and control by gentle means the tumultuous movements of the body and limbs.
2. To support the strength of the organism by abundant nourishment.
3. To allay apprehension and irritability, and to induce sleep.
4. To watch for and to treat complications as they may arise.

We shall consider the above headings in detail:—

1. On admission into the hospital the patient is allotted a separate room, if possible, or a quiet corner in the general ward, and is put on a large water-bed filled with hot water, over which the draw-sheet is kept tightly stretched (a point of great importance) to minimise chafing. The body and limbs are examined for bed-sores, and the elbows, knees, and ankles are covered with sheets of cotton-wool, bandaged firmly. In a recent severe case a small soft feather pillow was fastened by means of webbing straps over each elbow, to the great relief of the patient, whose joints were showing signs of sores.

The head of the bed is padded with folded blankets encased in sheeting as high as the patient can reach in the recumbent position.

Special care is taken to prevent chafing by frequent adjustment of the bed clothes, and by "tucking-up" tightly, especially across the knees. A soft flannel shirt is put on if the movements are not very violent. The feet are sometimes tied together and fastened to the foot of the bed by flannel bandages over the swathings above-mentioned.

In some cases, where there is a constant tendency to roll out of bed, and the patient is strong, a contrivance known as "bed boards" is used, composed of thin deal planks about fourteen inches in depth, fastened by a series of hooks upon the iron-work of the bedstead round the sides, so as to form, with the bed, a shallow trough in which the patient lies. Folded blankets and pillows are used to pad the boards and prevent any part of the patient coming in contact with the bare wood.

The hair is cut short.

An evaporating lotion composed of nitric acid and spirits of nitrous ether, in the proportion of one minim of the former to an ounce of the latter, is rubbed over all the parts subject to much friction, at least every three hours. The back is carefully dried with a soft towel after washing, and starch powder used freely. A wet pack is occasionally applied with quieting effect, especially if the temperature rises.

2. *Nourishment* is given in large quantities, and is often taken well, but sometimes there is trouble with its administration, owing to difficulty in deglutition.

For a girl fifteen years old the following dietary is a fair sample: Milk, two or three pints; beef-tea, one pint (made in the proportion of a pound of raw beef to a pint of water); four to six eggs, one or two lemons, made into lemonade. Nourishment is given every half-hour while the patient is awake.

If there is difficulty in feeding, nutrient enemata are administered, composed of varying quantities of eggs, milk, and beef-tea (peptonised).

The feeding cup has the spout protected by a piece of indiarubber tubing drawn over it, a very necessary precaution, for on one occasion not only was the spout bitten off, but nearly swallowed.

The following terse dictum of Dr. Hughlings Jackson, when discussing the treatment of the degrees of severity of the choreic symptoms, is broadly carried out: (1) Food (abundant nourishment), (2) alcohol, (3) chloral (narcotics), (4) chloroform (anæsthetics).

Alcohol is freely given if indications of flagging manifest themselves, and is considered to rank in the second line of defence. Brandy is used more frequently than the other forms of alcohol. The quantity taken is sometimes as much as six or eight ounces in twenty-four hours. The Mistura Spiritus Vini Gallici of the British Pharmacopœia is often taken more readily than brandy and milk.

3. To allay apprehension and irritability is considered to be of vital importance. Hysterical symptoms, temper, delusions, spectral hallucinations, or even delirium being not infrequently found in varying combinations, care is taken to keep away all sources of excitement. The relatives of the patient are excluded. The room is darkened, the bed surrounded with screens, and all lights shaded. Frequent sponging of the face and extremities with cold or warm water is found to be very comforting. The mouth is frequently washed with borax and glycerine or with lemon juice, and sordes removed from lips and teeth.

Two special nurses are appointed to tend the patient, one on day and the other on night duty. On no account is the patient left alone while the acute symptoms last.

Sleep is induced by the use of drugs when milder measures have failed. Choral hydrate, bromide of potash, sulphonal, and paraldehyde have been used at various times. Paraldehyde has recently been found very useful when given in drachm doses every hour until sleep follows. In a recent case several hours' sound sleep were induced after the eighth dose.

If the violence of the movements continues unabated, with sleeplessness, increased pulse rate, and signs of exhaustion in spite of these remedies, there is no hesitation in administering chloroform. The method of administration is as follows. A piece of lint saturated with the anæsthetic is placed over the patient's face until the movements cease and the breathing becomes quiet and regular, then the lint is removed until there are signs of awakening. By this means the patient is kept quiet for seven or eight consecutive hours. Half an hour's sleep is often produced by the inhalation of a few drops of chloroform, after the preliminary anæsthesia. The resident medical officers each take duty as anæsthetist for "spells" of an hour. Sleep so obtained is considered to be of great value. Occasionally chloroform has been given for three or four nights in succession.

4. The temperature is taken every four hours, if possible. The heart is examined daily. Constipation is corrected by laxatives or enemata. The presence or absence of pregnancy, carious teeth, worms, or other sources of irritation are noted carefully.

In conclusion, we should say that the treatment of acute chorea is considered mainly a matter of skilled and tender nursing. The majority of cases recover after one or more relapses, but convalescence is slow, often many months elapsing before mental and bodily vigour are regained.

